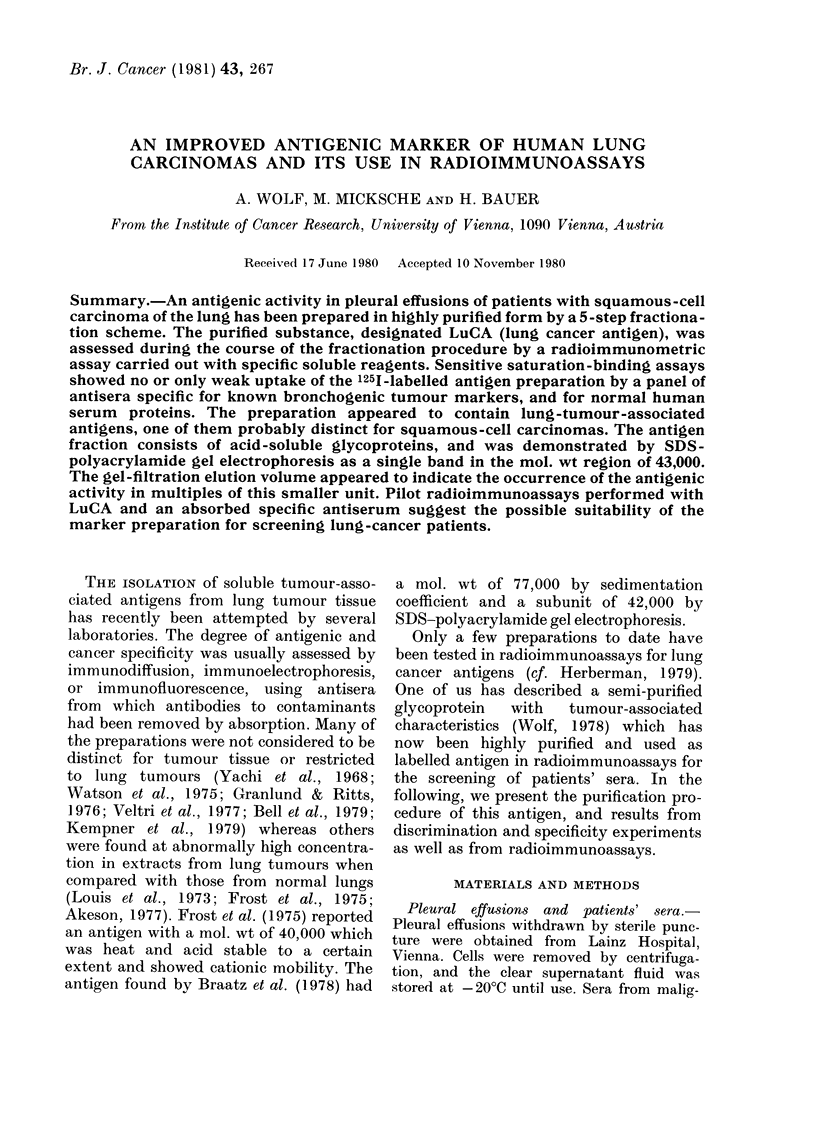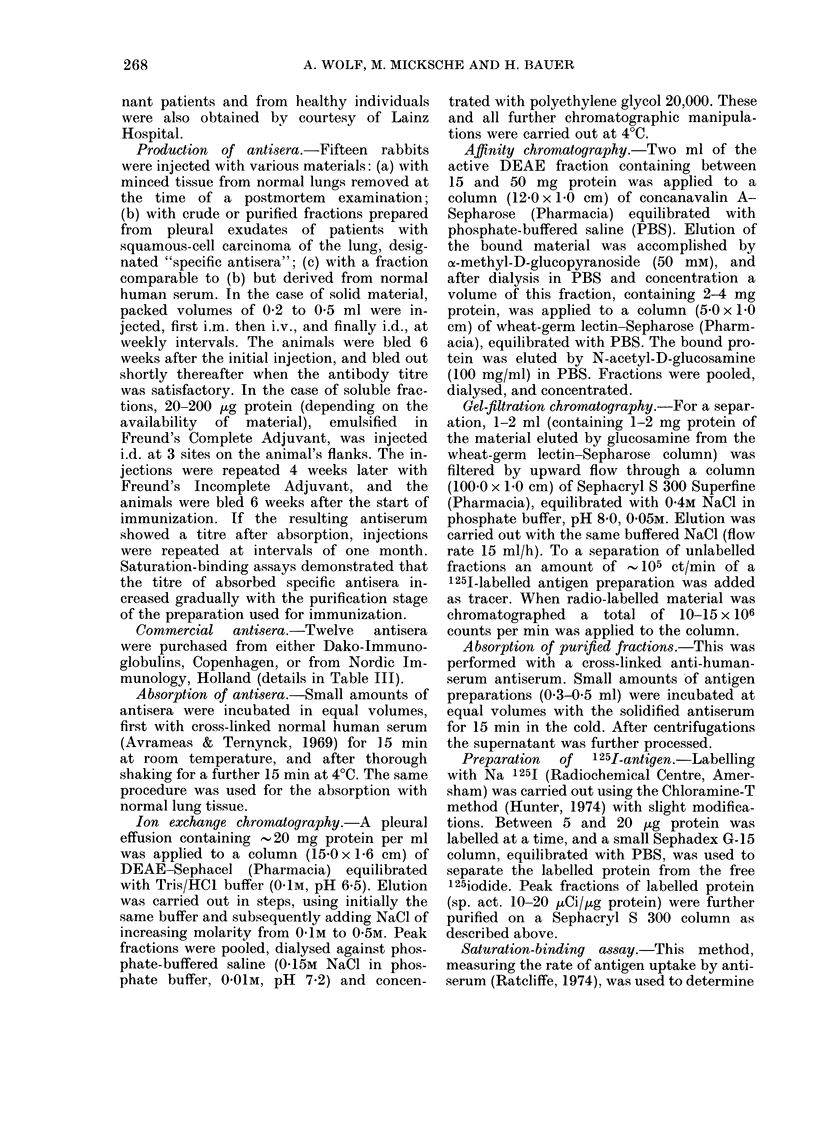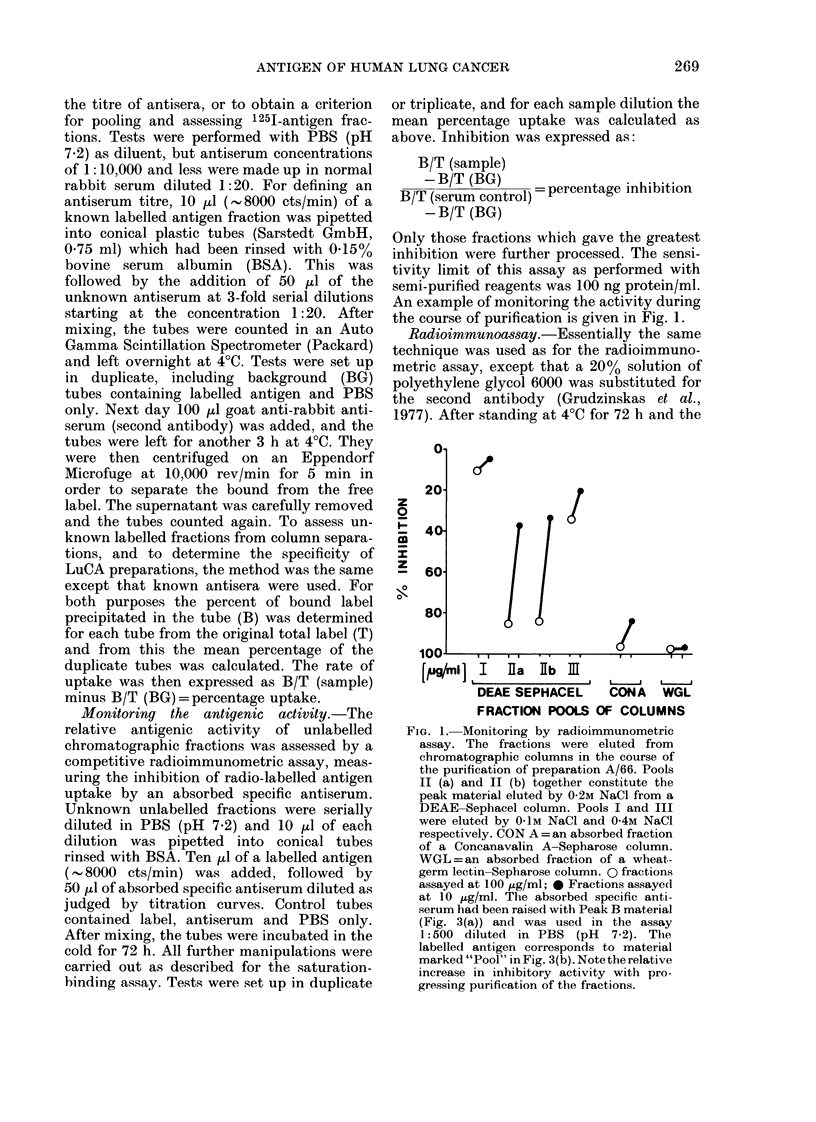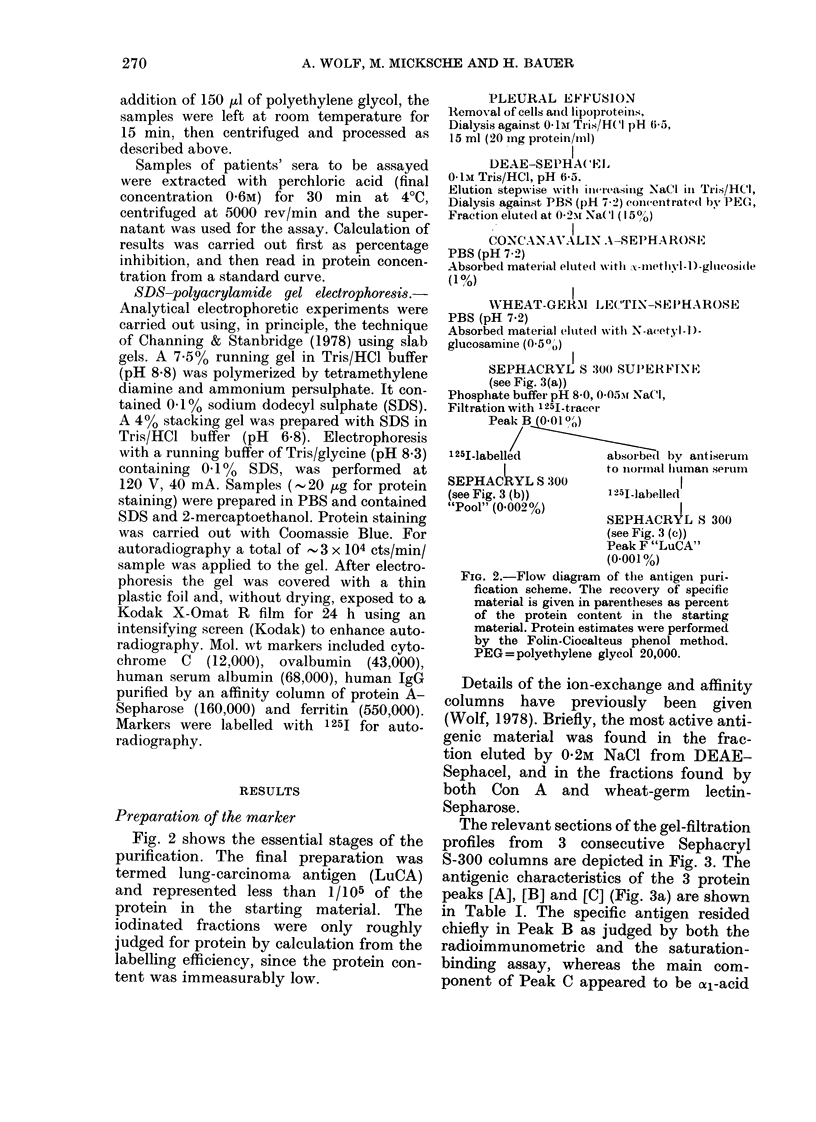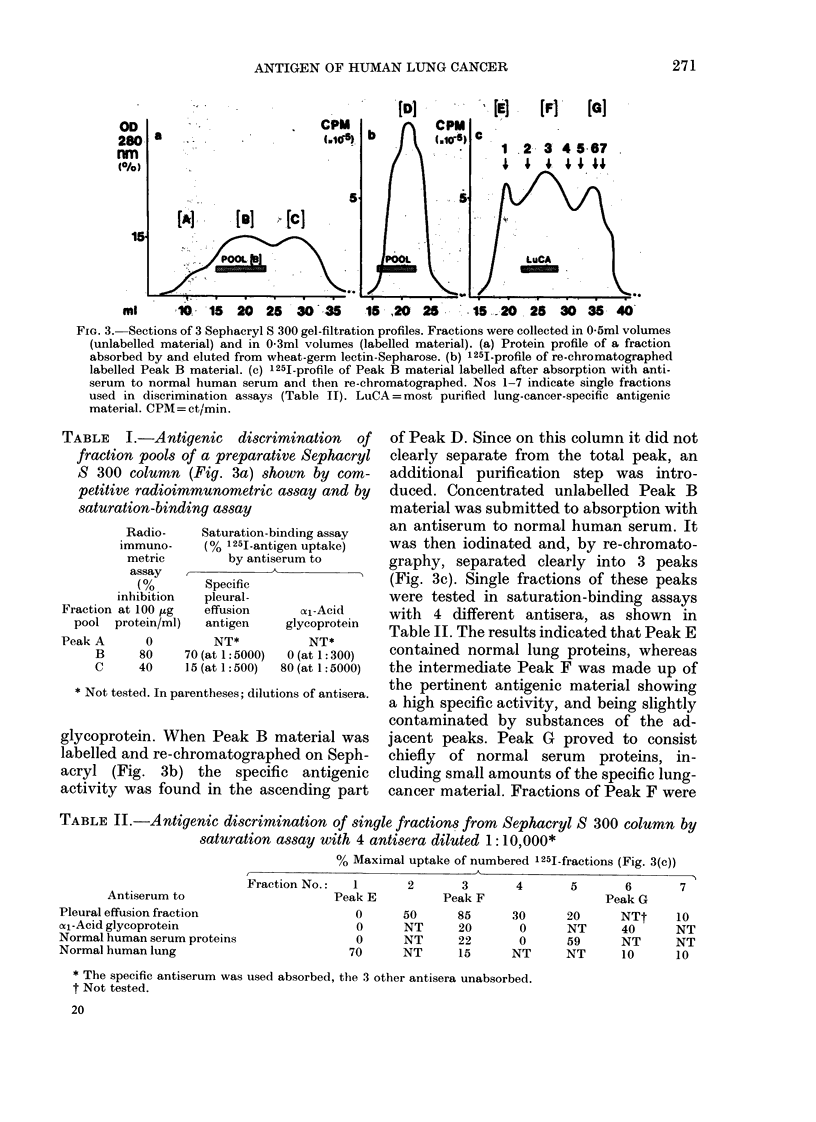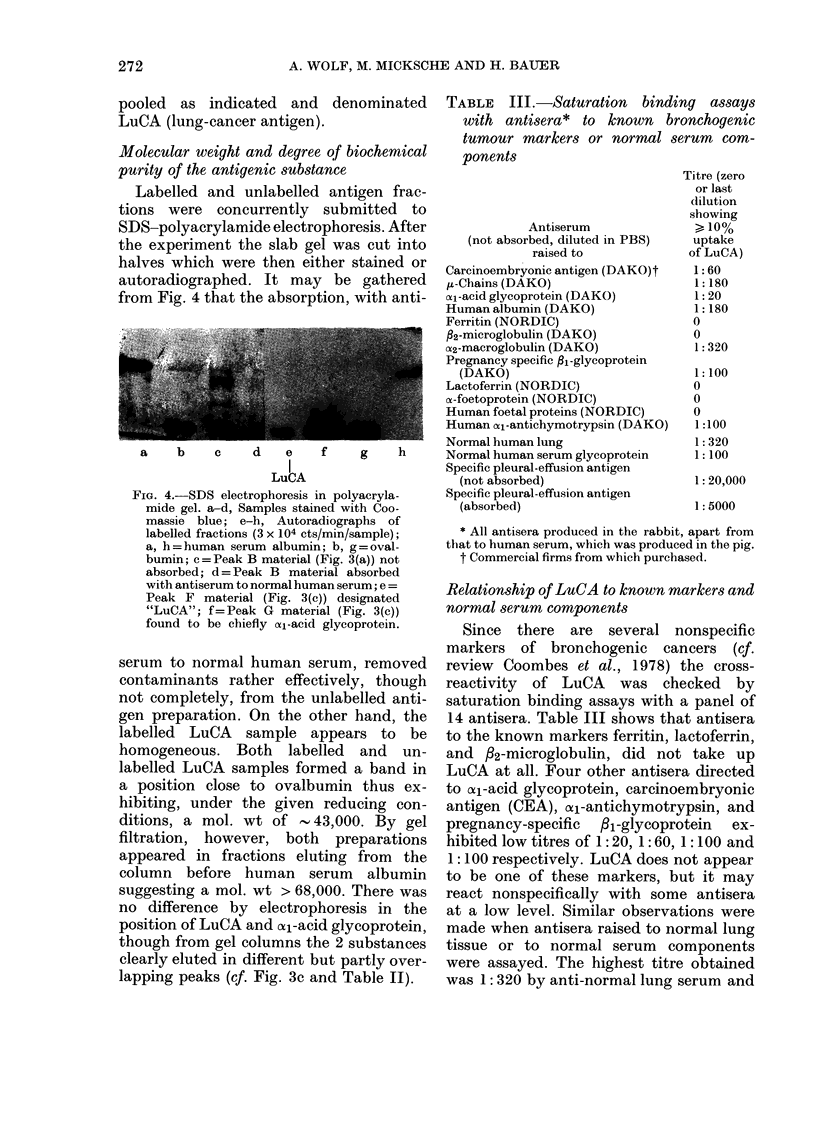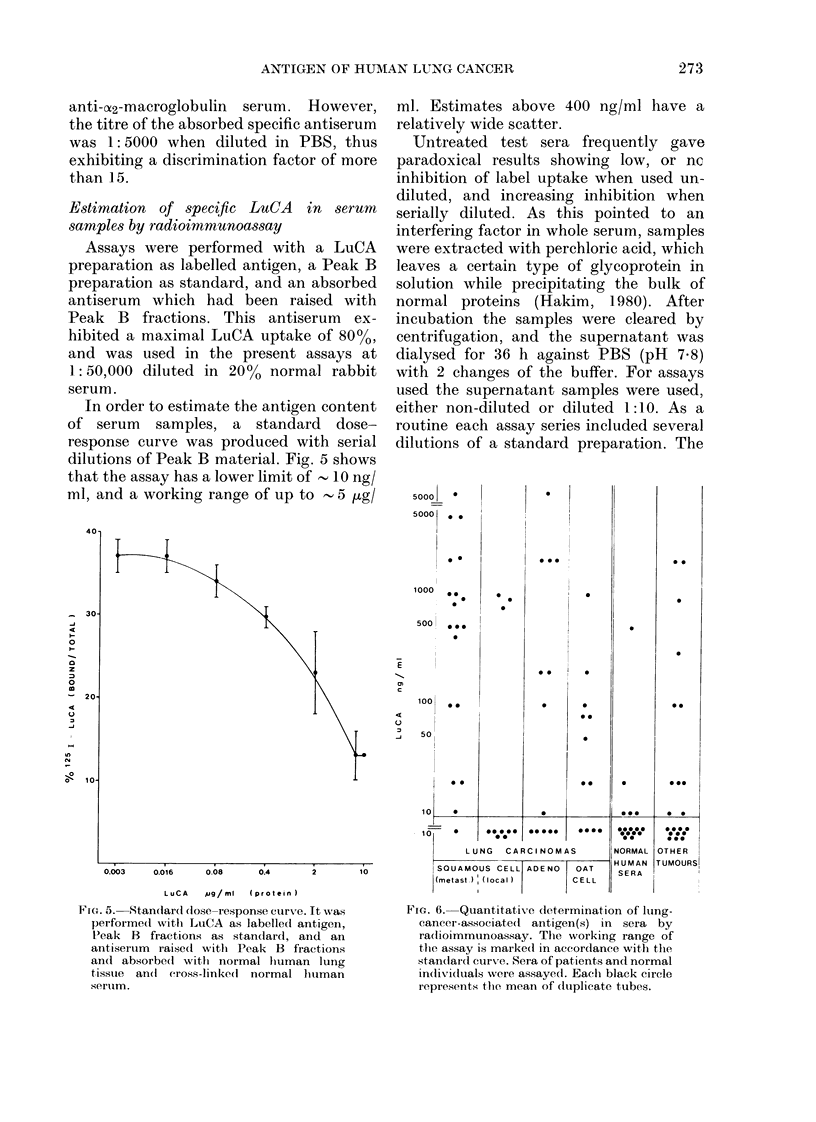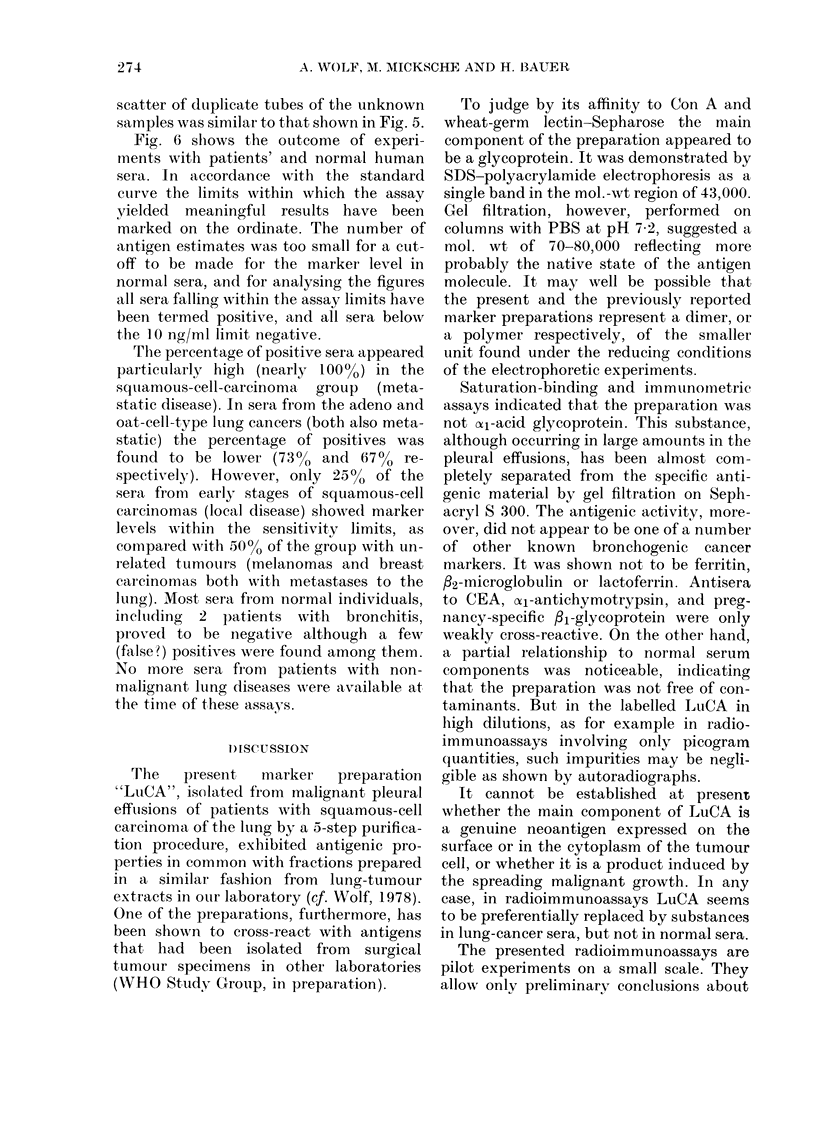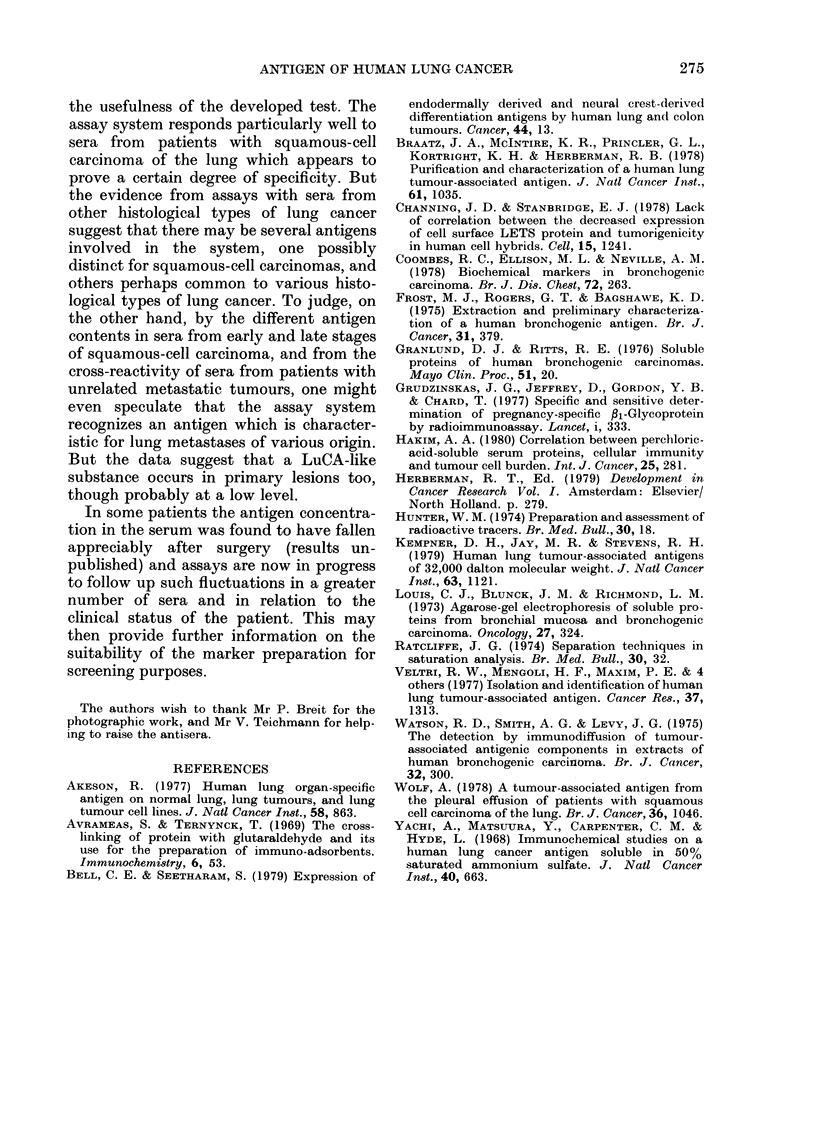# An improved antigenic marker of human lung carcinomas and its use in radioimmunoassays.

**DOI:** 10.1038/bjc.1981.44

**Published:** 1981-03

**Authors:** A. Wolf, M. Micksche, H. Bauer

## Abstract

**Images:**


*Br. J. Cancer* (1981) **43**, 267

### AN IMPROVED ANTIGENIC MARKER OF HUMAN LUNG CARCINOMAS AND ITS USE IN RADIOIMMUNOASSAYS

A. WOLF, M. MICKSCHE AND H. BAUER

*From the Institute of Cancer Research, University of Vienna,* 1090 *Vienna, Austria*

Received 17 June 1980 Accepted 10 November 1980

**Summary.—An antigenic activity in pleural effusions of patients with squamous-cell carcinoma of the lung has been prepared in highly purified form by a 5-step fractionation scheme. The purified substance, designated LuCA (lung cancer antigen), was assessed during the course of the fractionation procedure by a radioimmunometric assay carried out with specific soluble reagents. Sensitive saturation-binding assays showed no or only weak uptake of the $^{125}$I-labelled antigen preparation by a panel of antisera specific for known bronchogenic tumour markers, and for normal human serum proteins. The preparation appeared to contain lung-tumour-associated antigens, one of them probably distinct for squamous-cell carcinomas. The antigen fraction consists of acid-soluble glycoproteins, and was demonstrated by SDS-polyacrylamide gel electrophoresis as a single band in the mol. wt region of 43,000. The gel-filtration elution volume appeared to indicate the occurrence of the antigenic activity in multiples of this smaller unit. Pilot radioimmunoassays performed with LuCA and an absorbed specific antiserum suggest the possible suitability of the marker preparation for screening lung-cancer patients.**

THE ISOLATION of soluble tumour-associated antigens from lung tumour tissue has recently been attempted by several laboratories. The degree of antigenic and cancer specificity was usually assessed by immunodiffusion, immunoelectrophoresis, or immunofluorescence, using antisera from which antibodies to contaminants had been removed by absorption. Many of the preparations were not considered to be distinct for tumour tissue or restricted to lung tumours (Yachi *et al.*, 1968; Watson *et al.*, 1975; Granlund & Ritts, 1976; Veltri *et al.*, 1977; Bell *et al.*, 1979; Kempner *et al.*, 1979) whereas others were found at abnormally high concentration in extracts from lung tumours when compared with those from normal lungs (Louis *et al.*, 1973; Frost *et al.*, 1975; Akeson, 1977). Frost *et al.* (1975) reported an antigen with a mol. wt of 40,000 which was heat and acid stable to a certain extent and showed cationic mobility. The antigen found by Braatz *et al.* (1978) had a mol. wt of 77,000 by sedimentation coefficient and a subunit of 42,000 by SDS–polyacrylamide gel electrophoresis.

Only a few preparations to date have been tested in radioimmunoassays for lung cancer antigens (*cf.* Herberman, 1979). One of us has described a semi-purified glycoprotein with tumour-associated characteristics (Wolf, 1978) which has now been highly purified and used as labelled antigen in radioimmunoassays for the screening of patients' sera. In the following, we present the purification procedure of this antigen, and results from discrimination and specificity experiments as well as from radioimmunoassays.

### MATERIALS AND METHODS

*Pleural effusions and patients' sera.*—Pleural effusions withdrawn by sterile puncture were obtained from Lainz Hospital, Vienna. Cells were removed by centrifugation, and the clear supernatant fluid was stored at −20°C until use. Sera from malignant patients and from healthy individuals were also obtained by courtesy of Lainz Hospital.

*Production of antisera.*—Fifteen rabbits were injected with various materials: (a) with minced tissue from normal lungs removed at the time of a postmortem examination; (b) with crude or purified fractions prepared from pleural exudates of patients with squamous-cell carcinoma of the lung, designated "specific antisera"; (c) with a fraction comparable to (b) but derived from normal human serum. In the case of solid material, packed volumes of 0·2 to 0·5 ml were injected, first i.m. then i.v., and finally i.d., at weekly intervals. The animals were bled 6 weeks after the initial injection, and bled out shortly thereafter when the antibody titre was satisfactory. In the case of soluble fractions, 20–200 μg protein (depending on the availability of material), emulsified in Freund's Complete Adjuvant, was injected i.d. at 3 sites on the animal's flanks. The injections were repeated 4 weeks later with Freund's Incomplete Adjuvant, and the animals were bled 6 weeks after the start of immunization. If the resulting antiserum showed a titre after absorption, injections were repeated at intervals of one month. Saturation-binding assays demonstrated that the titre of absorbed specific antisera increased gradually with the purification stage of the preparation used for immunization.

*Commercial antisera.*—Twelve antisera were purchased from either Dako-Immunoglobulins, Copenhagen, or from Nordic Immunology, Holland (details in Table III).

*Absorption of antisera.*—Small amounts of antisera were incubated in equal volumes, first with cross-linked normal human serum (Avrameas & Ternynck, 1969) for 15 min at room temperature, and after thorough shaking for a further 15 min at 4°C. The same procedure was used for the absorption with normal lung tissue.

*Ion exchange chromatography.*—A pleural effusion containing ~20 mg protein per ml was applied to a column (15·0 × 1·6 cm) of DEAE–Sephacel (Pharmacia) equilibrated with Tris/HCl buffer (0·1M, pH 6·5). Elution was carried out in steps, using initially the same buffer and subsequently adding NaCl of increasing molarity from 0·1M to 0·5M. Peak fractions were pooled, dialysed against phosphate-buffered saline (0·15M NaCl in phosphate buffer, 0·01M, pH 7·2) and concentrated with polyethylene glycol 20,000. These and all further chromatographic manipulations were carried out at 4°C.

*Affinity chromatography.*—Two ml of the active DEAE fraction containing between 15 and 50 mg protein was applied to a column (12·0 × 1·0 cm) of concanavalin A–Sepharose (Pharmacia) equilibrated with phosphate-buffered saline (PBS). Elution of the bound material was accomplished by α-methyl-D-glucopyranoside (50 mM), and after dialysis in PBS and concentration a volume of this fraction, containing 2–4 mg protein, was applied to a column (5·0 × 1·0 cm) of wheat-germ lectin–Sepharose (Pharmacia), equilibrated with PBS. The bound protein was eluted by N-acetyl-D-glucosamine (100 mg/ml) in PBS. Fractions were pooled, dialysed, and concentrated.

*Gel-filtration chromatography.*—For a separation, 1–2 ml (containing 1–2 mg protein of the material eluted by glucosamine from the wheat-germ lectin–Sepharose column) was filtered by upward flow through a column (100·0 × 1·0 cm) of Sephacryl S 300 Superfine (Pharmacia), equilibrated with 0·4M NaCl in phosphate buffer, pH 8·0, 0·05M. Elution was carried out with the same buffered NaCl (flow rate 15 ml/h). To a separation of unlabelled fractions an amount of $\sim 10^5$ ct/min of a $^{125}$I-labelled antigen preparation was added as tracer. When radio-labelled material was chromatographed a total of $10–15 \times 10^6$ counts per min was applied to the column.

*Absorption of purified fractions.*—This was performed with a cross-linked anti-human-serum antiserum. Small amounts of antigen preparations (0·3–0·5 ml) were incubated at equal volumes with the solidified antiserum for 15 min in the cold. After centrifugations the supernatant was further processed.

*Preparation of $^{125}$I-antigen.*—Labelling with Na $^{125}$I (Radiochemical Centre, Amersham) was carried out using the Chloramine-T method (Hunter, 1974) with slight modifications. Between 5 and 20 μg protein was labelled at a time, and a small Sephadex G-15 column, equilibrated with PBS, was used to separate the labelled protein from the free $^{125}$iodide. Peak fractions of labelled protein (sp. act. 10–20 μCi/μg protein) were further purified on a Sephacryl S 300 column as described above.

*Saturation-binding assay.*—This method, measuring the rate of antigen uptake by antiserum (Ratcliffe, 1974), was used to determine the titre of antisera, or to obtain a criterion for pooling and assessing $^{125}$I-antigen fractions. Tests were performed with PBS (pH 7·2) as diluent, but antiserum concentrations of 1:10,000 and less were made up in normal rabbit serum diluted 1:20. For defining an antiserum titre, 10 $\mu$l ($\sim$8000 cts/min) of a known labelled antigen fraction was pipetted into conical plastic tubes (Sarstedt GmbH, 0·75 ml) which had been rinsed with 0·15% bovine serum albumin (BSA). This was followed by the addition of 50 $\mu$l of the unknown antiserum at 3-fold serial dilutions starting at the concentration 1:20. After mixing, the tubes were counted in an Auto Gamma Scintillation Spectrometer (Packard) and left overnight at 4°C. Tests were set up in duplicate, including background (BG) tubes containing labelled antigen and PBS only. Next day 100 $\mu$l goat anti-rabbit antiserum (second antibody) was added, and the tubes were left for another 3 h at 4°C. They were then centrifuged on an Eppendorf Microfuge at 10,000 rev/min for 5 min in order to separate the bound from the free label. The supernatant was carefully removed and the tubes counted again. To assess unknown labelled fractions from column separations, and to determine the specificity of LuCA preparations, the method was the same except that known antisera were used. For both purposes the percent of bound label precipitated in the tube (B) was determined for each tube from the original total label (T) and from this the mean percentage of the duplicate tubes was calculated. The rate of uptake was then expressed as B/T (sample) minus B/T (BG) = percentage uptake.

*Monitoring the antigenic activity.*—The relative antigenic activity of unlabelled chromatographic fractions was assessed by a competitive radioimmunometric assay, measuring the inhibition of radio-labelled antigen uptake by an absorbed specific antiserum. Unknown unlabelled fractions were serially diluted in PBS (pH 7·2) and 10 $\mu$l of each dilution was pipetted into conical tubes rinsed with BSA. Ten $\mu$l of a labelled antigen ($\sim$8000 cts/min) was added, followed by 50 $\mu$l of absorbed specific antiserum diluted as judged by titration curves. Control tubes contained label, antiserum and PBS only. After mixing, the tubes were incubated in the cold for 72 h. All further manipulations were carried out as described for the saturation-binding assay. Tests were set up in duplicate or triplicate, and for each sample dilution the mean percentage uptake was calculated as above. Inhibition was expressed as:

$$\frac{\text{B/T (sample)} - \text{B/T (BG)}}{\text{B/T (serum control)} - \text{B/T (BG)}} = \text{percentage inhibition}$$

Only those fractions which gave the greatest inhibition were further processed. The sensitivity limit of this assay as performed with semi-purified reagents was 100 ng protein/ml. An example of monitoring the activity during the course of purification is given in Fig. 1.

*Radioimmunoassay.*—Essentially the same technique was used as for the radioimmunometric assay, except that a 20% solution of polyethylene glycol 6000 was substituted for the second antibody (Grudzinskas *et al.*, 1977). After standing at 4°C for 72 h and the

FIG. 1.—Monitoring by radioimmunometric assay. The fractions were eluted from chromatographic columns in the course of the purification of preparation A/66. Pools II (a) and II (b) together constitute the peak material eluted by 0·2M NaCl from a DEAE–Sephacel column. Pools I and III were eluted by 0·1M NaCl and 0·4M NaCl respectively. CON A = an absorbed fraction of a Concanavalin A–Sepharose column. WGL = an absorbed fraction of a wheat-germ lectin–Sepharose column. ○ fractions assayed at 100 $\mu$g/ml; ● Fractions assayed at 10 $\mu$g/ml. The absorbed specific antiserum had been raised with Peak B material (Fig. 3(a)) and was used in the assay 1:500 diluted in PBS (pH 7·2). The labelled antigen corresponds to material marked "Pool" in Fig. 3(b). Note the relative increase in inhibitory activity with progressing purification of the fractions.

addition of 150 $\mu$l of polyethylene glycol, the samples were left at room temperature for 15 min, then centrifuged and processed as described above.

Samples of patients' sera to be assayed were extracted with perchloric acid (final concentration 0·6M) for 30 min at 4°C, centrifuged at 5000 rev/min and the supernatant was used for the assay. Calculation of results was carried out first as percentage inhibition, and then read in protein concentration from a standard curve.

*SDS-polyacrylamide gel electrophoresis.*—Analytical electrophoretic experiments were carried out using, in principle, the technique of Channing & Stanbridge (1978) using slab gels. A 7·5% running gel in Tris/HCl buffer (pH 8·8) was polymerized by tetramethylene diamine and ammonium persulphate. It contained 0·1% sodium dodecyl sulphate (SDS). A 4% stacking gel was prepared with SDS in Tris/HCl buffer (pH 6·8). Electrophoresis with a running buffer of Tris/glycine (pH 8·3) containing 0·1% SDS, was performed at 120 V, 40 mA. Samples ($\sim$20 $\mu$g for protein staining) were prepared in PBS and contained SDS and 2-mercaptoethanol. Protein staining was carried out with Coomassie Blue. For autoradiography a total of $\sim 3 \times 10^4$ cts/min/sample was applied to the gel. After electrophoresis the gel was covered with a thin plastic foil and, without drying, exposed to a Kodak X-Omat R film for 24 h using an intensifying screen (Kodak) to enhance autoradiography. Mol. wt markers included cytochrome C (12,000), ovalbumin (43,000), human serum albumin (68,000), human IgG purified by an affinity column of protein A-Sepharose (160,000) and ferritin (550,000). Markers were labelled with $^{125}$I for autoradiography.

### RESULTS

### *Preparation of the marker*

Fig. 2 shows the essential stages of the purification. The final preparation was termed lung-carcinoma antigen (LuCA) and represented less than $1/10^5$ of the protein in the starting material. The iodinated fractions were only roughly judged for protein by calculation from the labelling efficiency, since the protein content was immeasurably low.

PLEURAL EFFUSION

Removal of cells and lipoproteins,

Dialysis against 0·1M Tris/HCl pH 6·5,

15 ml (20 mg protein/ml)

DEAE-SEPHACEL

0·1M Tris/HCl, pH 6·5.

Elution stepwise with increasing NaCl in Tris/HCl,

Dialysis against PBS (pH 7·2) concentrated by PEG,

Fraction eluted at 0·2M NaCl (15%)

CONCANAVALIN A-SEPHAROSE
PBS (pH 7·2)
Absorbed material eluted with $\alpha$-methyl-D-glucoside (1%)

WHEAT-GERM LECTIN-SEPHAROSE
PBS (pH 7·2)
Absorbed material eluted with N-acetyl-D-glucosamine (0·5%)

SEPHACRYL S 300 SUPERFINE

(see Fig. 3(a))

Phosphate buffer pH 8·0, 0·05M NaCl,

Filtration with $^{125}$I-tracer

Peak B (0·01%)

$^{125}$I-labelled

SEPHACRYL S 300

(see Fig. 3 (b))

"Pool" (0·002%)

absorbed by antiserum to normal human serum

$^{125}$I-labelled

SEPHACRYL S 300

(see Fig. 3 (c))

Peak F "LuCA"

(0·001%)

FIG. 2.—Flow diagram of the antigen purification scheme. The recovery of specific material is given in parentheses as percent of the protein content in the starting material. Protein estimates were performed by the Folin-Ciocalteus phenol method. PEG = polyethylene glycol 20,000.

Details of the ion-exchange and affinity columns have previously been given (Wolf, 1978). Briefly, the most active antigenic material was found in the fraction eluted by 0·2M NaCl from DEAE-Sephacel, and in the fractions found by both Con A and wheat-germ lectin-Sepharose.

The relevant sections of the gel-filtration profiles from 3 consecutive Sephacryl S-300 columns are depicted in Fig. 3. The antigenic characteristics of the 3 protein peaks [A], [B] and [C] (Fig. 3a) are shown in Table I. The specific antigen resided chiefly in Peak B as judged by both the radioimmunometric and the saturation-binding assay, whereas the main component of Peak C appeared to be $\alpha_1$-acid

FIG. 3.—Sections of 3 Sephacryl S 300 gel-filtration profiles. Fractions were collected in 0·5ml volumes (unlabelled material) and in 0·3ml volumes (labelled material). (a) Protein profile of a fraction absorbed by and eluted from wheat-germ lectin-Sepharose. (b) $^{125}$I-profile of re-chromatographed labelled Peak B material. (c) $^{125}$I-profile of Peak B material labelled after absorption with antiserum to normal human serum and then re-chromatographed. Nos 1–7 indicate single fractions used in discrimination assays (Table II). LuCA = most purified lung-cancer-specific antigenic material. CPM = ct/min.

TABLE I.—*Antigenic discrimination of fraction pools of a preparative Sephacryl S 300 column (Fig. 3a) shown by competitive radioimmunometric assay and by saturation-binding assay*

| Fraction pool | Radio-immuno-metric assay (% inhibition at 100 μg protein/ml) | Saturation-binding assay (% $^{125}$I-antigen uptake) by antiserum to: Specific pleural-effusion antigen | $\alpha_1$-Acid glycoprotein |
|---|---|---|---|
| Peak A | 0 | NT* | NT* |
| B | 80 | 70 (at 1:5000) | 0 (at 1:300) |
| C | 40 | 15 (at 1:500) | 80 (at 1:5000) |

* Not tested. In parentheses; dilutions of antisera.

glycoprotein. When Peak B material was labelled and re-chromatographed on Sephacryl (Fig. 3b) the specific antigenic activity was found in the ascending part of Peak D. Since on this column it did not clearly separate from the total peak, an additional purification step was introduced. Concentrated unlabelled Peak B material was submitted to absorption with an antiserum to normal human serum. It was then iodinated and, by re-chromatography, separated clearly into 3 peaks (Fig. 3c). Single fractions of these peaks were tested in saturation-binding assays with 4 different antisera, as shown in Table II. The results indicated that Peak E contained normal lung proteins, whereas the intermediate Peak F was made up of the pertinent antigenic material showing a high specific activity, and being slightly contaminated by substances of the adjacent peaks. Peak G proved to consist chiefly of normal serum proteins, including small amounts of the specific lung-cancer material. Fractions of Peak F were

TABLE II.—*Antigenic discrimination of single fractions from Sephacryl S 300 column by saturation assay with* 4 *antisera diluted* 1:10,000*

| Antiserum to | % Maximal uptake of numbered $^{125}$I-fractions (Fig. 3(c)) — Fraction No.: 1 Peak E | 2 | 3 Peak F | 4 | 5 | 6 Peak G | 7 |
|---|---|---|---|---|---|---|---|
| Pleural effusion fraction | 0 | 50 | 85 | 30 | 20 | NT† | 10 |
| $\alpha_1$-Acid glycoprotein | 0 | NT | 20 | 0 | NT | 40 | NT |
| Normal human serum proteins | 0 | NT | 22 | 0 | 59 | NT | NT |
| Normal human lung | 70 | NT | 15 | NT | NT | 10 | 10 |

* The specific antiserum was used absorbed, the 3 other antisera unabsorbed.
† Not tested.

20

pooled as indicated and denominated LuCA (lung-cancer antigen).

*Molecular weight and degree of biochemical purity of the antigenic substance*

Labelled and unlabelled antigen fractions were concurrently submitted to SDS–polyacrylamide electrophoresis. After the experiment the slab gel was cut into halves which were then either stained or autoradiographed. It may be gathered from Fig. 4 that the absorption, with anti-

FIG. 4.—SDS electrophoresis in polyacrylamide gel. a–d, Samples stained with Coomassie blue; e–h, Autoradiographs of labelled fractions ($3 \times 10^4$ cts/min/sample); a, h = human serum albumin; b, g = ovalbumin; c = Peak B material (Fig. 3(a)) not absorbed; d = Peak B material absorbed with antiserum to normal human serum; e = Peak F material (Fig. 3(c)) designated "LuCA"; f = Peak G material (Fig. 3(c)) found to be chiefly $\alpha_1$-acid glycoprotein.

serum to normal human serum, removed contaminants rather effectively, though not completely, from the unlabelled antigen preparation. On the other hand, the labelled LuCA sample appears to be homogeneous. Both labelled and unlabelled LuCA samples formed a band in a position close to ovalbumin thus exhibiting, under the given reducing conditions, a mol. wt of ~43,000. By gel filtration, however, both preparations appeared in fractions eluting from the column before human serum albumin suggesting a mol. wt > 68,000. There was no difference by electrophoresis in the position of LuCA and $\alpha_1$-acid glycoprotein, though from gel columns the 2 substances clearly eluted in different but partly overlapping peaks (*cf.* Fig. 3c and Table II).

TABLE III.—*Saturation binding assays with antisera* to known bronchogenic tumour markers or normal serum components*

| Antiserum (not absorbed, diluted in PBS) raised to | Titre (zero or last dilution showing ⩾10% uptake of LuCA) |
|---|---|
| Carcinoembryonic antigen (DAKO)† | 1:60 |
| $\mu$-Chains (DAKO) | 1:180 |
| $\alpha_1$-acid glycoprotein (DAKO) | 1:20 |
| Human albumin (DAKO) | 1:180 |
| Ferritin (NORDIC) | 0 |
| $\beta_2$-microglobulin (DAKO) | 0 |
| $\alpha_2$-macroglobulin (DAKO) | 1:320 |
| Pregnancy specific $\beta_1$-glycoprotein (DAKO) | 1:100 |
| Lactoferrin (NORDIC) | 0 |
| $\alpha$-foetoprotein (NORDIC) | 0 |
| Human foetal proteins (NORDIC) | 0 |
| Human $\alpha_1$-antichymotrypsin (DAKO) | 1:100 |
| Normal human lung | 1:320 |
| Normal human serum glycoprotein | 1:100 |
| Specific pleural-effusion antigen (not absorbed) | 1:20,000 |
| Specific pleural-effusion antigen (absorbed) | 1:5000 |

* All antisera produced in the rabbit, apart from that to human serum, which was produced in the pig.
† Commercial firms from which purchased.

*Relationship of LuCA to known markers and normal serum components*

Since there are several nonspecific markers of bronchogenic cancers (*cf.* review Coombes *et al.*, 1978) the cross-reactivity of LuCA was checked by saturation binding assays with a panel of 14 antisera. Table III shows that antisera to the known markers ferritin, lactoferrin, and $\beta_2$-microglobulin, did not take up LuCA at all. Four other antisera directed to $\alpha_1$-acid glycoprotein, carcinoembryonic antigen (CEA), $\alpha_1$-antichymotrypsin, and pregnancy-specific $\beta_1$-glycoprotein exhibited low titres of 1:20, 1:60, 1:100 and 1:100 respectively. LuCA does not appear to be one of these markers, but it may react nonspecifically with some antisera at a low level. Similar observations were made when antisera raised to normal lung tissue or to normal serum components were assayed. The highest titre obtained was 1:320 by anti-normal lung serum and anti-$\alpha_2$-macroglobulin serum. However, the titre of the absorbed specific antiserum was 1:5000 when diluted in PBS, thus exhibiting a discrimination factor of more than 15.

*Estimation of specific LuCA in serum samples by radioimmunoassay*

Assays were performed with a LuCA preparation as labelled antigen, a Peak B preparation as standard, and an absorbed antiserum which had been raised with Peak B fractions. This antiserum exhibited a maximal LuCA uptake of 80%, and was used in the present assays at 1:50,000 diluted in 20% normal rabbit serum.

In order to estimate the antigen content of serum samples, a standard dose–response curve was produced with serial dilutions of Peak B material. Fig. 5 shows that the assay has a lower limit of ~10 ng/ml, and a working range of up to ~5 μg/ml. Estimates above 400 ng/ml have a relatively wide scatter.

Untreated test sera frequently gave paradoxical results showing low, or nc inhibition of label uptake when used undiluted, and increasing inhibition when serially diluted. As this pointed to an interfering factor in whole serum, samples were extracted with perchloric acid, which leaves a certain type of glycoprotein in solution while precipitating the bulk of normal proteins (Hakim, 1980). After incubation the samples were cleared by centrifugation, and the supernatant was dialysed for 36 h against PBS (pH 7·8) with 2 changes of the buffer. For assays used the supernatant samples were used, either non-diluted or diluted 1:10. As a routine each assay series included several dilutions of a standard preparation. The

FIG. 5.—Standard dose–response curve. It was performed with LuCA as labelled antigen, Peak B fractions as standard, and an antiserum raised with Peak B fractions and absorbed with normal human lung tissue and cross-linked normal human serum.

FIG. 6.—Quantitative determination of lung-cancer-associated antigen(s) in sera by radioimmunoassay. The working range of the assay is marked in accordance with the standard curve. Sera of patients and normal individuals were assayed. Each black circle represents the mean of duplicate tubes.

scatter of duplicate tubes of the unknown samples was similar to that shown in Fig. 5.

Fig. 6 shows the outcome of experiments with patients' and normal human sera. In accordance with the standard curve the limits within which the assay yielded meaningful results have been marked on the ordinate. The number of antigen estimates was too small for a cut-off to be made for the marker level in normal sera, and for analysing the figures all sera falling within the assay limits have been termed positive, and all sera below the 10 ng/ml limit negative.

The percentage of positive sera appeared particularly high (nearly 100%) in the squamous-cell-carcinoma group (metastatic disease). In sera from the adeno and oat-cell-type lung cancers (both also metastatic) the percentage of positives was found to be lower (73% and 67% respectively). However, only 25% of the sera from early stages of squamous-cell carcinomas (local disease) showed marker levels within the sensitivity limits, as compared with 50% of the group with unrelated tumours (melanomas and breast carcinomas both with metastases to the lung). Most sera from normal individuals, including 2 patients with bronchitis, proved to be negative although a few (false?) positives were found among them. No more sera from patients with non-malignant lung diseases were available at the time of these assays.

### DISCUSSION

The present marker preparation "LuCA", isolated from malignant pleural effusions of patients with squamous-cell carcinoma of the lung by a 5-step purification procedure, exhibited antigenic properties in common with fractions prepared in a similar fashion from lung-tumour extracts in our laboratory (*cf.* Wolf, 1978). One of the preparations, furthermore, has been shown to cross-react with antigens that had been isolated from surgical tumour specimens in other laboratories (WHO Study Group, in preparation).

To judge by its affinity to Con A and wheat-germ lectin–Sepharose the main component of the preparation appeared to be a glycoprotein. It was demonstrated by SDS–polyacrylamide electrophoresis as a single band in the mol.-wt region of 43,000. Gel filtration, however, performed on columns with PBS at pH 7·2, suggested a mol. wt of 70–80,000 reflecting more probably the native state of the antigen molecule. It may well be possible that the present and the previously reported marker preparations represent a dimer, or a polymer respectively, of the smaller unit found under the reducing conditions of the electrophoretic experiments.

Saturation-binding and immunometric assays indicated that the preparation was not $\alpha_1$-acid glycoprotein. This substance, although occurring in large amounts in the pleural effusions, has been almost completely separated from the specific antigenic material by gel filtration on Sephacryl S 300. The antigenic activity, moreover, did not appear to be one of a number of other known bronchogenic cancer markers. It was shown not to be ferritin, $\beta_2$-microglobulin or lactoferrin. Antisera to CEA, $\alpha_1$-antichymotrypsin, and pregnancy-specific $\beta_1$-glycoprotein were only weakly cross-reactive. On the other hand, a partial relationship to normal serum components was noticeable, indicating that the preparation was not free of contaminants. But in the labelled LuCA in high dilutions, as for example in radioimmunoassays involving only picogram quantities, such impurities may be negligible as shown by autoradiographs.

It cannot be established at present whether the main component of LuCA is a genuine neoantigen expressed on the surface or in the cytoplasm of the tumour cell, or whether it is a product induced by the spreading malignant growth. In any case, in radioimmunoassays LuCA seems to be preferentially replaced by substances in lung-cancer sera, but not in normal sera.

The presented radioimmunoassays are pilot experiments on a small scale. They allow only preliminary conclusions about the usefulness of the developed test. The assay system responds particularly well to sera from patients with squamous-cell carcinoma of the lung which appears to prove a certain degree of specificity. But the evidence from assays with sera from other histological types of lung cancer suggest that there may be several antigens involved in the system, one possibly distinct for squamous-cell carcinomas, and others perhaps common to various histological types of lung cancer. To judge, on the other hand, by the different antigen contents in sera from early and late stages of squamous-cell carcinoma, and from the cross-reactivity of sera from patients with unrelated metastatic tumours, one might even speculate that the assay system recognizes an antigen which is characteristic for lung metastases of various origin. But the data suggest that a LuCA-like substance occurs in primary lesions too, though probably at a low level.

In some patients the antigen concentration in the serum was found to have fallen appreciably after surgery (results unpublished) and assays are now in progress to follow up such fluctuations in a greater number of sera and in relation to the clinical status of the patient. This may then provide further information on the suitability of the marker preparation for screening purposes.

The authors wish to thank Mr P. Breit for the photographic work, and Mr V. Teichmann for helping to raise the antisera.

### REFERENCES

Akeson, R. (1977) Human lung organ-specific antigen on normal lung, lung tumours, and lung tumour cell lines. *J. Natl Cancer Inst.*, **58**, 863.

Avrameas, S. & Ternynck, T. (1969) The cross-linking of protein with glutaraldehyde and its use for the preparation of immuno-adsorbents. *Immunochemistry*, **6**, 53.

Bell, C. E. & Seetharam, S. (1979) Expression of endodermally derived and neural crest-derived differentiation antigens by human lung and colon tumours. *Cancer*, **44**, 13.

Braatz, J. A., McIntire, K. R., Princler, G. L., Kortright, K. H. & Herberman, R. B. (1978) Purification and characterization of a human lung tumour-associated antigen. *J. Natl Cancer Inst.*, **61**, 1035.

Channing, J. D. & Stanbridge, E. J. (1978) Lack of correlation between the decreased expression of cell surface LETS protein and tumorigenicity in human cell hybrids. *Cell*, **15**, 1241.

Coombes, R. C., Ellison, M. L. & Neville, A. M. (1978) Biochemical markers in bronchogenic carcinoma. *Br. J. Dis. Chest*, **72**, 263.

Frost, M. J., Rogers, G. T. & Bagshawe, K. D. (1975) Extraction and preliminary characterization of a human bronchogenic antigen. *Br. J. Cancer*, **31**, 379.

Granlund, D. J. & Ritts, R. E. (1976) Soluble proteins of human bronchogenic carcinomas. *Mayo Clin. Proc.*, **51**, 20.

Grudzinskas, J. G., Jeffrey, D., Gordon, Y. B. & Chard, T. (1977) Specific and sensitive determination of pregnancy-specific $\beta_1$-Glycoprotein by radioimmunoassay. *Lancet*, i, 333.

Hakim, A. A. (1980) Correlation between perchloric-acid-soluble serum proteins, cellular immunity and tumour cell burden. *Int. J. Cancer*, **25**, 281.

Herberman, R. T., Ed. (1979) *Development in Cancer Research Vol. I.* Amsterdam: Elsevier/North Holland. p. 279.

Hunter, W. M. (1974) Preparation and assessment of radioactive tracers. *Br. Med. Bull.*, **30**, 18.

Kempner, D. H., Jay, M. R. & Stevens, R. H. (1979) Human lung tumour-associated antigens of 32,000 dalton molecular weight. *J. Natl Cancer Inst.*, **63**, 1121.

Louis, C. J., Blunck, J. M. & Richmond, L. M. (1973) Agarose-gel electrophoresis of soluble proteins from bronchial mucosa and bronchogenic carcinoma. *Oncology*, **27**, 324.

Ratcliffe, J. G. (1974) Separation techniques in saturation analysis. *Br. Med. Bull.*, **30**, 32.

Veltri, R. W., Mengoli, H. F., Maxim, P. E. & 4 others (1977) Isolation and identification of human lung tumour-associated antigen. *Cancer Res.*, **37**, 1313.

Watson, R. D., Smith, A. G. & Levy, J. G. (1975) The detection by immunodiffusion of tumour-associated antigenic components in extracts of human bronchogenic carcinoma. *Br. J. Cancer*, **32**, 300.

Wolf, A. (1978) A tumour-associated antigen from the pleural effusion of patients with squamous cell carcinoma of the lung. *Br. J. Cancer*, **36**, 1046.

Yachi, A., Matsuura, Y., Carpenter, C. M. & Hyde, L. (1968) Immunochemical studies on a human lung cancer antigen soluble in 50% saturated ammonium sulfate. *J. Natl Cancer Inst.*, **40**, 663.